# Acknowledgement to Reviewers of *Life* in 2017

**DOI:** 10.3390/life8010002

**Published:** 2018-01-12

**Authors:** Life Editorial Office

**Affiliations:** MDPI AG, St. Alban-Anlage 66, 4052 Basel, Switzerland

Peer review is an essential part in the publication process, ensuring that *Life* maintains high quality standards for its published papers. In 2017, a total of 51 papers were published in the journal. Thanks to the cooperation of our reviewers, the median time to first decision was 21 days and the median time to publication was 51 days. The editors would like to express their sincere gratitude to the following reviewers for their time and dedication in 2017:

Aaron EngelhartLiam M. LongoAaron S. BurtonLinda McGownAdam KunLisa SteinAddy ProssLoren WilliamsAlexander BolshoyLuc JaegerAna CrnkovicLukasz DziewitAndre BrackMarc TesseraAndrés de la EscosuraMarc T. FacciottiAndrew J. SurmanMarie-Christine, MaurelArmen MulkidjanianMarkus RalserArturo BecerraMartha A. GroverBalázs KönnyűMassimo Di GiulioBarry SavilleMatthew PasekBonnie BaxterMatthew PownerBrian J. CaffertyMichael BlaberBrian R. FrancisMichael HechtCarl DevitoMichael RussellCarl TrindleMichael SchweizerChandra WickramasingheMichele FioreCharles LiottaMike Dyall-SmithCharles Jr. CarterMikhail M. Vorob’evChristian MayerMin GuoChristoph A. WeberMV JoseChristophe LavelleNam-Joon ChoChunliang LiuNediljko BudisaConstantinos StathopoulosNiles LehmanCynthia M. MortonNils HolmDave SpeijerNobuhiko SuematsuDavid PennyPasquale StanoDavid S. RossPatrick O’DonoghueDennis GörlichPaul ClarkeDiego Luis GonzalezPaul HiggsDino MorasPeter StrazewskiDuncan ForganPeter UnrauEliana Beltrán-PardoPeter WaldeElias ChatzitheodoridisPeter R. WillsEmanuele ZonaroRafal M. WieczorekEnver Cagri IzguRaffaele SaladinoFalcinelli StefanoRainer MerklFengjie SunRalf MöllerFilipa L. SousaRichard EgelFilippo CascheraRichard P. FahlmanGabriel ZentnerRob M. De GraafGeoff WildRobert PascalGeorge FoxRobert Root-BernsteinGeorgy KarevRomeu GuimaraesGreg SpringsteenRosanna del GaudioGregory FournierRussell F. DoolittleHarold S. BernhardtSandra PizzarelloHelen HansmaSatoshi AkanumaHervé SeligmannSheref MansyHodaka FujiiShin-ichi YokoboriHubert Dominique BeckerSimone GianneriniHussein KaddourSolveig TosiHyman HartmanSreejith RamakrishnanIgor ShuryakStefan FoxJack T. O’Malley-JamesStuart BartlettJason RaymondSzymon WasikJiann-Ruey HongTakahiro HohsakaJiri ŠponerTetsuo KushiroJoanne K. HobbsTetsushi SakumaJohn YinThomas GeorgelinJordi PiñeroThomas SimonsonJuan Manuel García-RuizThomas C. BoothbyKaren BellThomas M. OrlandoKelly SheppardTony JiaKenji IkeharaUlrich MullerKenneth A. DillVeronica VaidaKensaku SakamotoWilliam BainsKepa Ruiz-MirazoWilliam H. McClainKoichiro MatsunoYoshihiro FurukawaKumar Saurabh SinghYu-Fen ZhaoKunio KawamuraA.V. KorotayevLeonardo Sorci


